# From Redox Imbalance to Tissue Injury: Insights Into Antidepressant Drug Amitriptyline Effects on Salivary Glands

**DOI:** 10.1002/cbf.70189

**Published:** 2026-03-01

**Authors:** Cristian dos Santos Pereira, Deiweson Souza‐Monteiro, Yago Gecy de Sousa Né, Jorddy Neves da Cruz, Vinicius Ruan Neves dos Santos, Everton Luiz Pompeu Varela, Sandro Percário, Leonardo Oliveira Bittencourt, Antonio Hernandes Chaves‐Neto, Rafael Rodrigues Lima

**Affiliations:** ^1^ Laboratory of Functional and Structural Biology, Institute of Biological Sciences Federal University of Pará (UFPA) Belém Pará Brazil; ^2^ Oxidative Stress Research Laboratory, Institute of Biological Sciences Federal University of Para (UFPA) Belém Pará Brazil; ^3^ Department of Basic Sciences School of Dentistry of Araçatuba Universidade Estadual Paulista (UNESP) Araçatuba São Paulo Brazil

**Keywords:** amitriptyline, morphometry, oxidative stress, salivary glands

## Abstract

This study aimed to investigate the effects of amitriptyline administration on the salivary glands and saliva of rats. Twenty‐eight male Wistar rats (60 days old) were divided into two groups (*n* = 14 per group): control and amitriptyline‐treated (10 mg/kg/day for 30 days). After the treatment period, saliva samples induced by pilocarpine were collected to analyze total protein concentration, amylase activity, and antioxidant capacity, while salivary glands were harvested for assessments of oxidative stress markers and morphological changes. Amitriptyline increased total protein and decreased amylase activity in saliva, with no change in the Trolox‐equivalent antioxidant capacity (TEAC). The drug triggered oxidative stress in both glands by the decrease in TEAC concentration and increased lipid peroxidation. Morphometric analysis showed that amitriptyline increased the total area of stroma and decreased the ductal area in both glands. In the submandibular gland, acinar area was reduced as well. These findings suggest that amitriptyline‐salivary gland dysfunction is associated with oxidative imbalance, morphometric, and alterations in saliva composition, contributing to a broader understanding of amitriptyline's adverse effects.

## Summary

The study aimed to evaluate the effects of chronic amitriptyline administration on the salivary glands, given the doubts about its biochemical, morphological, and functional repercussions. Exposure to the drug reduced amylase activity, modulated salivary protein content, and induced oxidative stress in the submandibular and parotid glands, which also showed significant morphological changes. These findings highlight the physiological and histological effects of amitriptyline, contributing to the understanding of its adverse mechanisms. In addition, they provide a basis for future research on the systemic effects of the drug, the development of therapeutic strategies to reduce its harm, and the safer use of tricyclic antidepressants in clinical practice.

## 1 Introduction

Amitriptyline is a tricyclic antidepressant approved by the Food and Drug Administration (FDA), commonly prescribed for the management of chronic pain and affective disorders [1], with therapeutic effect attributed to its ability to inhibit the reuptake of serotonin and noradrenaline [2]. However, it also produces off‐target activity, acting on histaminergic, muscarinic, and other receptors [3]. Some indications are not approved by the FDA, being used off‐label to treat diseases and conditions, such as fibromyalgia, diabetic neuropathy, and migraine prophylaxis [4]. Despite this, amitriptyline has been an effective therapeutic in several disease states and disorders, but it has also been a worrying source of toxicity and side effects [5].

Tachycardia, constipation, and confusion are some of the side effects observed on the use of amitriptyline [5]. Among the side effects, dry mouth is one of the most frequently reported [6]. Evidence suggests that, in addition to the central mechanisms of inhibitory action on the reuptake of serotonin and norepinephrine, peripheral effects may contribute to salivary hypofunction, as salivary glands possess the ability to uptake and secrete amitriptyline [7, 8]. In addition, experimental studies have demonstrated that amitriptyline produces harmful effects on salivary glands [9, 10, 11].

Amitriptyline has been related to induce oxidative stress and mitochondrial dysfunction in various organs. It has been associated to increased lipid peroxidation in liver and lung of mice [12]. Oxidative stress may be one of the mechanisms associated to morphological impairments in salivary glands as evidence indicates that systemic drugs, including sibutramine and benznidazole, can trigger oxidative imbalance, and such effects have been linked to subsequent morphometric alterations in glandular tissue. However, the current literature about amitriptyline and salivary glands lacks methodological robustness to elucidate the mechanisms involved in this scenario. Notably, Elsharkawy and Alhazzazi [10] focused solely on histological alterations and postulated the involvement of oxidative stress without performing biochemical analyses to substantiate its involvement. Additionally, the study by Yu [9] employed high‐dose amitriptyline administration via osmotic minipumps, a nonconventional route, alongside salivary stimulation through isoproterenol injection and parasympathetic nerve stimulation. These approaches diverge significantly from clinically relevant administration routes and current stimulation methods, such as the use of pilocarpine employed in our work. Consequently, the findings from these models limit the understanding of amitriptyline relation with salivary glands highlighting a critical gap in the literature.

Salivary glands are exocrine organs specialized in the production and secretion of saliva, a fluid essential for digestion, lubrication, antimicrobial defense, and oral mucosal integrity. Their architecture consists of serous and mucous acini, an extensive ductal system, myoepithelial cells, and dense vascularization, features that support intense protein synthesis and continuous exocytosis [13]. This elevated metabolic demand, combined with a high concentration of mitochondria and exposure to circulating substances, may predispose glandular tissue to reactive oxygen species (ROS) generation. As a consequence, salivary glands may be especially vulnerable to oxidative stress and to the intracellular accumulation of drugs or lipophilic compounds, leading to morphofunctional disturbances [14]. Therefore, this study aimed to evaluate the effects of chronic administration of amitriptyline on the salivary glands with the hypothesis that the administration of this drug in therapeutic doses equivalent to those used in humans induces oxidative and morphological changes in glandular tissue, resulting in modifications in salivary composition and function in rats.

## 2 Materials and Methods

### 2.1 Animals and Experimental Groups

All experiments were performed following the Guidelines for the Care and Use of Laboratory Animals [15] and reported following the Animal Research: Reports of In Vivo Experiments (ARRIVE) [16] after authorization from the Ethics Committee of Animal Use from the Federal University of Pará (CEUA‐UFPA) (Protocol N° 5458260821). Twenty‐eight male albino Wistar rats of the *Rattus norvegicus* species obtained from the Evandro Chagas Institute, with body mass between 130 and 150 g and 60 days old were used. The animals were housed in plastic cages appropriate to the species. During the housing period, the animals were fed with balanced pelleted food (Presence, Neovia, Brazil) and water ad libitum. They remained at a temperature of 25°C, in a dark/light cycle of 12 h.

The sample size was calculated using G*Power software (version 3.1.9.2), employing the test family “*t* tests” and the statistical test “Means: Difference between two independent means (two groups).” The calculation was based on a reference study with a similar experimental design [17], considering an effect size *d* of 1.45, an alpha error probability of 0.05, and a power (1 − *β*) of 0.80. Based on these parameters, the analysis indicated a minimum of seven animals per group.

In total, 28 rats were simple randomized into 2 groups with 14 animals each: control and amitriptyline. No previous criteria were used to allocate the animals. The exposed group received, via intragastric gavage, 10 mg/kg/day of amitriptyline hydrochloride (Sigma‐Aldrich, St. Louis, Missouri, USA, CAS No. 549‐18‐8) diluted in distilled water for 30 days, while the control group received only distilled water following the same protocol during the same period. The dose of amitriptyline was selected based on its therapeutic equivalence in humans, adjusted for rats using the allometric scaling methodology [18]. This calculation considers metabolic and physiological differences between species, applying the formula: Dose in rats (mg/kg) = Dose in humans (mg/kg)/Conversion factor (6.2). The dose of 10 mg/kg corresponds to the allometrically converted therapeutic dose in humans, as described in previous studies [19, 20]. The animals were weighted weekly to adjust the dose, if necessary.

Then, each group was subdivided into two subgroups, based on the type of tissue collection and analysis: nonperfused (seven animals) and perfused (seven animals). In the nonperfused, the parotid and submandibular salivary glands were collected, as well as saliva samples. In the perfused group, it was only collected samples of parotid and submandibular salivary glands. The nonperfused samples of salivary glands were analyzed for the following biochemical parameters: Trolox‐equivalent antioxidant capacity (TEAC), thiobarbituric acid reactive substances (TBARS), and reduced glutathione (GSH), while the saliva samples were submitted to TEAC, amylase activity, and total proteins. The perfused samples were submitted to histomorphometric analysis. Perfusion was applied only to samples destined for histology, as it preserves tissue architecture and improves sectioning, staining, and microscopic visualization. Because perfusate solutions may interfere with biochemical reactions and dilute endogenous molecules, nonperfused glands were instead used for biochemical assays. Therefore, separate animals were allocated for each type of analysis. No exclusion of animals was made. Cristian dos Santos Pereira was aware of the experiment stages. The authors worked on different analyses of the work.

### 2.2 Total Saliva Collection

Twenty‐four hours after the end of the experimental period, the nonperfused animals were anesthetized intraperitoneally with ketamine hydrochloride (90 mg/kg) and xylazine hydrochloride (9 mg/kg) [21]. After confirmation of total loss of corneal reflexes, salivation was stimulated with pilocarpine (1 mg/kg, i.p. Sigma‐Aldrich, St. Louis, Missouri, USA, CAS No. 54‐71‐7), and total saliva samples were collected under refrigeration in Eppendorf tubes, counting 5 min after the first drop falls into the tube. The samples were centrifuged at 1114*g* for 5 min, the supernatant was separated and stored at −80°C for subsequent analysis.

### 2.3 Euthanasia and Collection of Salivary Glands

Following total saliva collection, the animals were euthanized by exsanguination. Subsequently, samples of the submandibular and parotid salivary glands were collected, washed in saline solution, immediately frozen in liquid nitrogen, and stored at −80°C for further analysis. Regarding the animals for perfused tissue collection, the perfusion and exsanguination followed this protocol: after anesthesia, following the same procedure previously described, an incision was made in the left ventricle to insert a cannula, while the right atrium was opened to allow the perfusion fluids and blood to exit. After this, a saline solution (0.9% NaCl) containing heparin (1000 IU/mL) was administered to remove the circulating blood and prevent coagulation. This process was carried out until the outflow fluid showed a clear color, followed by the perfusion of 4% diluted formaldehyde. Then, the submandibular and parotid glands were collected for histomorphometric analysis.

### 2.4 Total Protein Concentration Assay and Amylase Activity

The determination of total protein levels in total saliva was used, which followed the method proposed by Bradford [22], in which the proteins bind to the Coomassie brilliant blue dye and form a blue compound with maximum absorbance at 595 nm, with unit in g/dL. In addition, amylase activity was evaluated using amylase colorimetric test that uses the modified Caraway method [23]. For this, we use the Bioclin Kit (Bioclin, K046‐1, Belo Horizonte, Brazil). The absorbances of the samples were measured in a spectrophotometer at 660 nm. The results of the amylase activity were expressed in U/dL.

### 2.5 Oxidative Biochemistry Assays

Salivary glands samples were thawed and resuspended in Tris‐HCl (20 mmol/L, pH 7.4) at 4°C and subsequently sonicated (concentration ~1 g/mL). The lysate was used to investigate GSH, TEAC, and TBARS levels: these parameters were then analyzed in salivary glands. Total saliva samples were used for TEAC analysis.

#### 2.5.1 TEAC

The assessment of TEAC levels was tested evaluating the ability to reduce the radical 2,2′‐azino‐bis (3‐ethylbenzothiazoline‐6‐sulfonic acid—ABTS). The samples were prepared following the method proposed by Miller et al. [24] and adapted by Re et al. [25]. Initially, a stock solution of ABTS (7.0 mM) (Sigma‐Aldrich A1888, St. Louis, MO, USA) was prepared in 0.1 M phosphate buffer (pH 7.4). Subsequently, the ABTS⁺• cation radical was generated by reacting the ABTS solution with potassium persulfate (K_2_S_2_O_8_, 2.45 mM) (Sigma‐Aldrich 60490) at a 1:1 (v/v) ratio. The mixture was kept protected from light for 12–16 h at room temperature, allowing complete oxidation and stable radical formation. After that, the samples were analyzed by spectrophotometry read at 734 nm for 5 min. The unit of measurement used for TEAC was determined in µmol/L. For the salivary glands, the values are presented as µmol of Trolox equivalents per gram of tissue (µmol TE/g). For saliva, the values are expressed as µmol of Trolox equivalents per mL (µmol TE/mL).

#### 2.5.2 GSH

In this method, the ability of glutathione (present in the sample) in reducing 5,5‐dithiobis‐2‐nitrobenzoic acid (DTNB; Sigma‐Aldrich, St. Louis, MO, USA) to nitrobenzoic acid is tested. The determination of GSH concentrations was performed according to what was proposed by Ellman [26]. For this, the samples were deproteinized with 2% trichloroacetic acid and the supernatant was collected for analysis after centrifugation at 1114*g* for 5 min. Initially, a 20 μL aliquot was taken from each sample and placed in a test tube containing 3 mL phosphate buffer and 20 μL of distilled water to perform the 1st reading of the sample (T0), then 100 μL of DTNB was added and after 3 min to perform the second reading of the sample (T3). The difference in absorbances (T3 – T0) is proportional to the concentration of GSH (Sigma‐Aldrich, St. Louis, MO, USA). The unit of measurement used was µmol/mL.

#### 2.5.3 TBARS

In this analysis, the test is carried out to determine the levels of lipid peroxidation through the dosage of TBARS levels. The method to evaluate lipid peroxidation was that of Kohn and Liversedge [27] and adapted by Percario et al. [28]. After the sonication step, the gland samples were added to a solution of malondialdehyde and thiobarbituric acid (TBA) (Sigma‐Aldrich, St. Louis, MO, USA) and washed in a water bath at 94°C for 1 h, where MDA is endogenously generated from lipid peroxidation and not added as a reagent. After stabilizing the temperature to room temperature, *n*‐butyl alcohol was added, followed by vortexing and centrifugation, the supernatant was subjected to spectrophotometric reading at 535 nm to obtain the TBARS data contained in the sample. The unit of measurement used was µmol/L. Biochemical analyses were performed without technical replicates; each sample was analyzed once.

### 2.6 Histomorphometric Analysis

After euthanasia and tissue fixation with 4% formaldehyde, samples of submandibular and parotid glands were postfixed in 4% formaldehyde for 24 h and then dehydrated in increasing concentrations of ethanol (70%, 80%, 90%, 100%), clarified in xylene, and embedded in paraffin. In this work, five sections were made per animal, and three fields per section were analyzed. Five‐micrometer sections, stained with hematoxylin and eosin, were obtained for morphometric analysis, taking into account the total area of stroma, acini, and ducts following the protocol already used by our research group, described in the work by Souza‐Monteiro et al. [29] and Fernandes et al. [30]. The analysis was performed using the Color Threshold function of the ImageJ software (version 2.4, Bethesda, Maryland, USA). Histological fields used for representative images were selected randomly from each slide to avoid subjective visual bias.

### 2.7 Statistical Analyses

**Figure 1:**
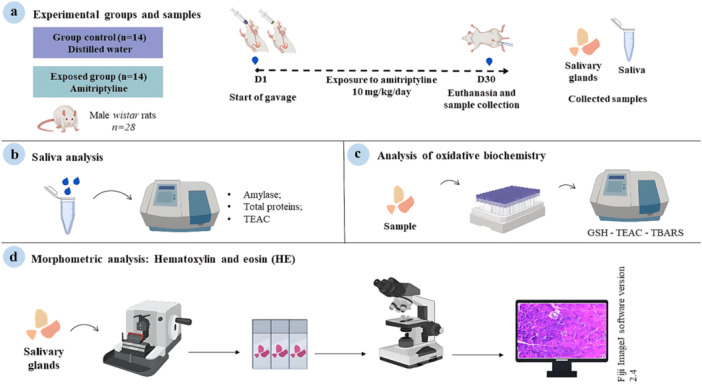
Methodological steps of the study. (a) Experimental groups, exposure to amitriptyline (10 mg/kg/day) for 30 days, euthanasia, and collection of biological samples (total saliva and salivary glands). (b) Total saliva analysis: amylase activity, total protein, and total capacity against peroxyl radicals (TEAC). (c) Oxidative biochemistry analysis of salivary glands: reduced glutathione (GSH) levels, TEAC, and thiobarbituric acid reactive substances (TBARS) levels. (d) Histomorphometric analysis of the salivary glands in HE.

The data obtained were tabulated and analyzed using GraphPad Prism software (version 8.0, Dotmatics) and Jamovi (version 2.6.17). The Shapiro–Wilk test was applied to determine the normality of the data. Parametric data were analyzed using the Student's *t*‐test, considering a *p* value of 0.05 as statistically significant. Results were expressed as mean ± standard error of the mean. In addition, multivariate analysis of variance (MANOVA) was performed in Jamovi to evaluate the combined effects of treatment on oxidative stress parameters (TEAC, TBARS, and GSH) in both the submandibular and parotid glands. Pillai's trace was used to determine overall significance. Pearson's correlation test was performed to assess the relationships between oxidative parameters, with Pearson's correlation coefficients calculated and interpreted for statistical significance. The test power was calculated using the difference groups' average with OpenEpi software (Version 3.01), considering the type I error of 5% and a power of 80% (for all values, see Table S1). Methodological steps are summarized in Figure 1.

## 3 Results

### 3.1 Exposure to Amitriptyline Reduced the Amylase Activity With Total Protein Content Modulation in Total Saliva Without Affect Its Antioxidant Capacity

**Figure 2:**
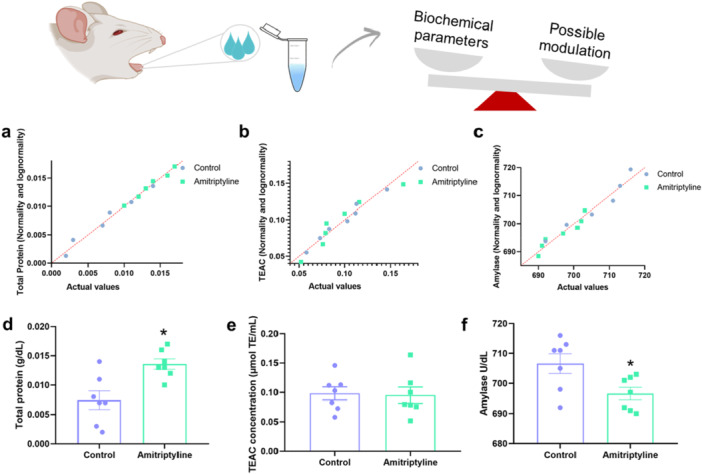
Effects of exposure to amitriptyline (10 mg/kg/day) for 30 days on salivary biochemical parameters in rats. Normality and lognormality tests are shown in (a–c). (d) Salivary amylase (U/dL), (e) total protein in total saliva (g/dL), and (f) total peroxyl radical scavenging capacity (μmol/L) (TEAC) concentration in total saliva. Results are expressed as mean ± SEM (*n* = 7). Student's *t*‐test (*p < 0.05).

Amitriptyline reduced the amylase activity in the exposed group (Control: 706.6 ± 3.3 U/dL; Amitriptyline: 696.6 ± 2.1 U/dL, *p* = 0.02). In addition, the total protein content in total saliva also differs between groups, with the exposed group showing increased concentration compared to the control (Control: 0.0074 ± 0.0015 g/dL; Amitriptyline: 0.0135 ± 0.0008 g/dL; *p* = 0.0056). However, no significant differences were found in salivary TEAC levels (Control: 0.098 ± 0.011 µmol/L; Amitriptyline: 0.095 ± 0.013 µmol/L, *p* = 0.86, Figure 2). The normality and lognormality data of the samples were also represented.

### 3.2 Amitriptyline Administration Is Associated to Oxidative Stress in Both Submandibular and Parotid Glands

**Figure 3:**
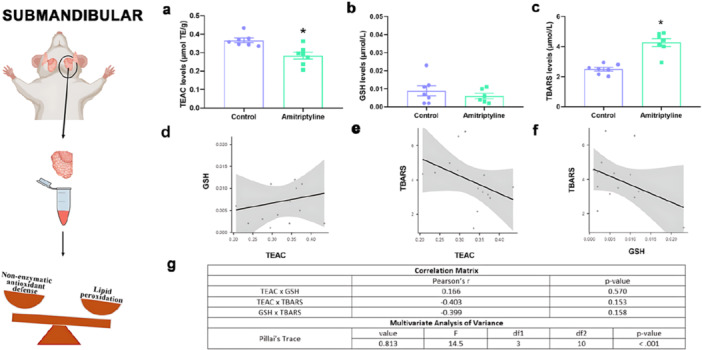
Effects of amitriptyline exposure (10 mg/kg/day) during 30 days in the oxidative biochemistry parameters of the submandibular gland of rats. (a) Total capacity against peroxyl radicals (TEAC) levels (μmol/L). (b) Glutathione reduced (GSH) levels (μmol/L). (c) Thiobarbituric acid reactive substances (TBARS) levels (μmol/L). Results are expressed as mean ± SEM (*n* = 7). Student's *t*‐test (*p < 0.05). (d) Correlation between TEAC and GSH concentrations. (e) Correlation between TEAC and TBARS concentrations. (f) Correlation between GSH and TBARS concentrations. (g) Multivariate analysis of variance results using Pillai's trace with value, *F* (test statistic), df1 (degree of freedom 1), df2 (degree of freedom 2), and *p* values.

In the submandibular glands, TEAC levels (Figure 3) showed a significant decrease in the amitriptyline‐treated group (0.284 ± 0.019 µmol/L) compared to the control group (0.366 ± 0.012 µmol/L, *p* = 0.037). However, our other analysis of reduced GSH showed no difference between the experimental groups (Control: 0.0088 ± 0.0027 µmol/L; Amitriptyline: 0.0052 ± 0.0014 µmol/L, *p* = 0.2799). The TBARS concentrations were significantly higher in the treated group (4.26 ± 0.25 µmol/L) when compared to the control group (2.49 ± 0.12 µmol/L, *p* < 0.0001), indicating oxidative stress.

Interestingly, the correlations for the submandibular gland did not show any correlations between TEAC and GSH (*r* = 0.166, *p* = 0.570), TEAC and TBARS (*r* = −0.403, *p* = 0.153), GSH and TBARS (*r* = −0.399, *p* = 0.158). However, the MANOVA showed a significant Pillai's trace value (0.813, *p* < 0.001) indicating statistically significant difference when all the parameters and groups are tested.

**Figure 4:**
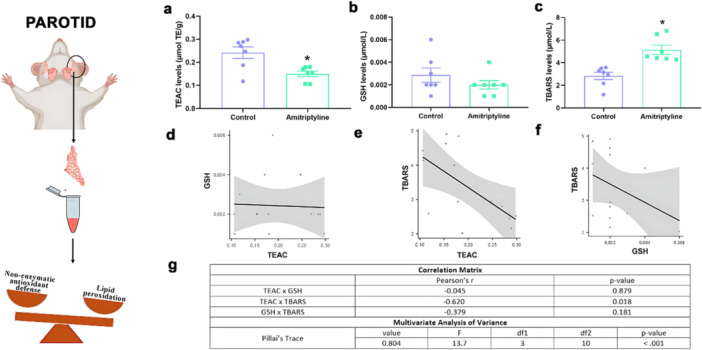
Effects of amitriptyline exposure (10 mg/kg/day) during 30 days in the oxidative biochemistry parameters of the parotid gland of rats. (a) Total capacity against peroxyl radicals (TEAC) levels (μmol/L). (b) Glutathione reduced (GSH) levels (μmol/L). (c) Thiobarbituric acid reactive substances (TBARS) levels (μmol/L). Results are expressed as mean ± SEM (*n* = 7). Student's *t*‐test (*p < 0.05). (d) Correlation between TEAC and GSH concentrations. (e) Correlation between TEAC and TBARS concentrations. (f) Correlation between GSH and TBARS concentrations. (g) Multivariate analysis of variance results using Pillai's trace with value, *F* (test statistic), df1 (degree of freedom 1), df2 (degree of freedom 2), and *p* values.

In the parotid glands, TEAC levels (Figure 4) showed a significant decrease in the amitriptyline‐treated group (0.150 ± 0.011 µmol/L) compared to the control group (0.242 ± 0.025 µmol/L, *p* = 0.0062). However, our analysis of reduced GSH showed no difference between groups (Control: 0.0028 ± 0.0006 µmol/L; Amitriptyline: 0.0020 ± 0.0003 µmol/L, *p* = 0.2679). The TBARS concentrations were significantly higher in the treated group (5.13 ± 0.40 µmol/L) when compared to the control group (2.82 ± 0.32 µmol/L, *p* = 0.0008) indicating oxidative stress.

In this gland, one correlation showed strong and statistically difference values, TEAC and TBARS (*r* = −0.620, *p *= 0.0018), but the other ones did not: GSH and TBARS (*r* = 0.379, *p* = 0.181), TEAC and GSH (*r* = −0.045, *p* = 0.879). The MANOVA results also revealed significant differences between the groups, with a Pillai's trace value of 0.804 (*p* < 0.001).

**Figure 5:**
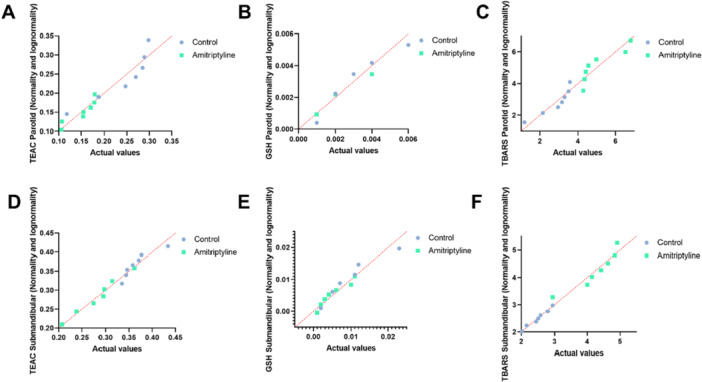
(A), (B), and (C) show the TEAC, GSH, and TBARS tests in the parotid glands. (D), (E), and (F) show the TEAC, GSH, and TBARS tests in the submandibular glands, considering a normal distribution according to the Shapiro–Wilk test. Seven animals were considered per group.

Figure 5 shows the normality and lognormality tests of the samples. All samples from both glands showed a normal distribution for the TEAC, GSH, and TBARS tests.

### 3.3 Amitriptyline‐Exposed Animals Showed Altered Morphological Parameters in Both Submandibular and Parotid Glands

**Figure 6:**
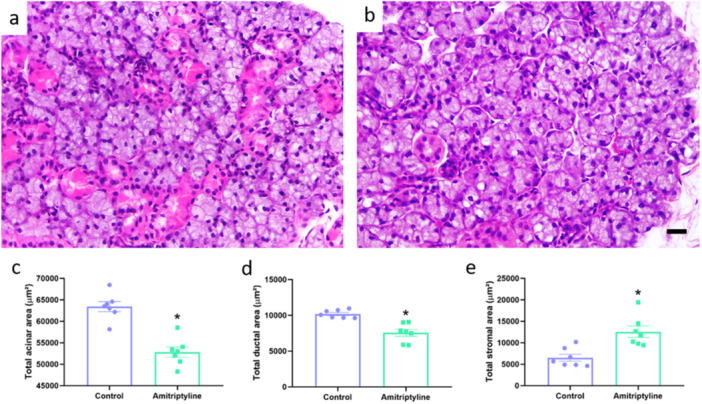
Effects of amitriptyline exposure (10 mg/kg/day) during 30 days in the morphometry of the submandibular gland of rats. Representative photomicrographs of the control (a) and amitriptyline (b) groups. (c) Total acinar area (µm^2^). (d) Total ductal area (µm^2^). (e) Total stromal area (µm^2^). Results are expressed as mean ± SEM (*n* = 7). Student's *t*‐test (*p < 0.05). Hematoxylin and eosin staining. Scale bar = 20 μm.

In the submandibular gland, the morphometric analysis showed that in the group that used amitriptyline there was a reduction in the total acinar area (Control: 63,404 ± 1163 µm^2^; Amitriptyline: 52,831 ± 1196 µm^2^; *p* < 0.0001; Figure 6), in the total ductal area (Control: 10,185 ± 212 µm^2^; Amitriptyline: 7546 ± 495 µm^2^; *p* = 0.0004). Interestingly, our data showed an increase in the total area stroma (Control: 6511 ± 817 µm^2^; Amitriptyline: 12,553 ± 1329 µm^2^; *p* = 0.022).

**Figure 7:**
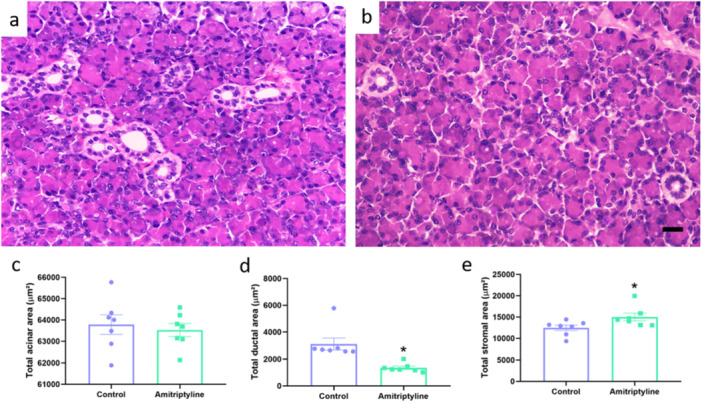
Effects of amitriptyline exposure (10 mg/kg/day) during 30 days in the morphometry of the parotid gland of rats. Representative photomicrographs of the control (a) and amitriptyline (b) groups. (c) Total acinar area (µm^2^). (d) Total ductal area (µm^2^). (e) Total stromal area (µm^2^). Results are expressed as mean ± SEM (*n* = 7). Student's *t*‐test (*p < 0.05). Hematoxylin and eosin staining. Scale bar = 20 μm. Magnification = 40×.

In the parotid gland, the morphometric analysis showed that in the group that used amitriptyline, there was a reduction in the total ductal area (Control: 3102 ± 446 µm^2^; Amitriptyline: 1352 ± 122 µm^2^; *p* = 0.0026) with an increase in the total area stroma (Control: 12,447 ± 651 µm^2^; Amitriptyline: 15,009 ± 899 µm^2^; *p* = 0.03). However, no difference was observed in the total acinar area (Control: 63,780 ± 460 µm^2^; Amitriptyline: 62,098 ± 1529 µm^2^; *p* = 0.31; Figure 7).

## 4 Discussion

Amitriptyline administration led to increased oxidative stress and morphological alterations in the salivary glands. Both the parotid and submandibular glands exhibited a significant reduction in TEAC levels alongside elevated TBARS concentrations, indicating oxidative imbalance. Morphologically, both glands displayed notable impairments in the stromal and ductal areas, with the submandibular gland also showing changes in the acinar area. These findings suggest that amitriptyline directly disrupts the oxidative balance and structural integrity of the salivary glands, potentially altering total saliva composition and function, as evidenced by changes in amylase activity and total protein content.

The parotid and submandibular glands are the primary organs responsible for saliva production, including ionic exchanges and secretion, which are essential for maintaining oral health. Together, these glands contribute ~95% of the total saliva produced, with the submandibular gland accounting for 60%–70% of this production [14, 30, 31]. Saliva plays a crucial role in maintaining oral cavity homeostasis, with functions that include buffering capacity, lubrication, and initiating digestion. Additionally, it is vital for enamel remineralization, especially during acidic challenges [32]. Given the importance of both glands for oral health, they were selected for this study as representatives of the primary salivary glands that perform these essential functions.

Our data show that amitriptyline treatment leads to increased oxidative stress, as evidenced by the significant decrease in TEAC and increase in TBARS concentrations in both the parotid and submandibular glands. The submandibular gland, in particular, showed more pronounced oxidative damage. These results are consistent with one clinical investigation reporting shifts in oxidative markers and increased lipid peroxidation during amitriptyline treatment, particularly during acute intoxication [33]. The significant oxidative changes observed in both glands support the idea that amitriptyline induces systemic dysfunction beyond the salivary glands.

In clinical settings, oxidative stress has been shown in psychiatric patients undergoing treatment with amitriptyline, as evidenced by a decline in antioxidant defenses, including reduced GSH concentration [34]. However, even though our study showed reduced nonenzymatic antioxidant capacity, the GSH concentration remain unaltered. This may suggest the involvement of alternative antioxidant pathways in salivary glands under these conditions. In this perspective, the increase in oxidative stress observed in the salivary glands following amitriptyline exposure is largely attributed to mitochondrial dysfunction in some studies, often related to tricyclic antidepressants [11, 35]. Amitriptyline can act as a nonspecific inhibitor of electron transport chain complexes [36], promoting electron leakage and excessive production of ROS. Consequently, the regeneration of antioxidants such as glutathione and thioredoxin tends to be limited, perpetuating a pro‐oxidant state. Mitochondrial dysfunction can also promote the opening of the mitochondrial permeability transition pore, leading to cytochrome c release into the cytosol and activation of the caspase cascade, culminating in cell death apoptosis [37].

The underlying mechanisms demonstrating that amitriptyline induces endoplasmic reticulum stress, characterized by the activation of the unfolded protein response, are based on the hypothesis that the sensors PERK, IRE1α, and ATF6 initiate adaptive responses aiming to temporarily reduce protein synthesis and increase chaperone expression [38, 39]. However, under persistent stress, these pathways would activate proapoptotic factors, such as CHOP, and inflammatory mediators via JNK and NF‐κB [40]. Furthermore, functional communication between mitochondria and the ER via mitochondria‐associated membranes exacerbates redox imbalance, calcium dysregulation, and apoptotic signaling [40]. In the context of salivary glands, chronic endoplasmic reticulum stress impairs salivary protein synthesis and secretion, suggesting a possible mechanism underlying the observed reduction in amylase activity and the paradoxical increase in total protein concentration, likely resulting from the nonselective secretion of stress‐related proteins and chaperones.

In terms of morphometry, the tissue of both salivary glands suffered alterations. Both glands showed a significant increase in stromal area with reduction in ductal area. However, submandibular gland also showed decrease in acinar area. These alterations can be directly associated with oxidative stress induced by amitriptyline, a mechanism already reported in other glandular systems. Chronic administration of amitriptyline has been shown to induce biochemical and structural changes in glandular tissues, such as reduced antioxidant capacity and increased lipoperoxidation [34, 41]. In salivary gland models, increased ROS have been linked to tissue damage, including stromal remodeling and loss of functional integrity [42]. These findings suggest that amitriptyline may induce glandular atrophy or tissue remodeling, particularly in the submandibular gland, driven by oxidative stress. Histological changes similar to these have been documented in other organs exposed to tricyclic antidepressants, such as cytotoxic and apoptotic effects observed in salivary glands [12].

The role of oxidative stress in histomorphological alterations is further supported by studies on similar drugs, such as venlafaxine and fluoxetine. These drugs are known to cause changes in glandular composition and reduced antioxidant capacity in salivary glands, resulting in increased lipoperoxidation markers and decreased glutathione levels [43]. These data corroborate the findings of this study, which demonstrated a relationship between increased oxidative stress and morphological alterations in salivary glands exposed to amitriptyline. It is important to clarify that the biochemical alterations observed in the salivary glands, specially the reduction in TEAC and the increase in TBARS, should not be interpreted as direct, linear determinants of the morphometric changes identified. TEAC represents a global, nonenzymatic antioxidant measure that reflects the tissue's integrated redox state, including potential compensatory or self‐regulatory adjustments within the antioxidant network. Thus, shifts in TEAC are more plausibly related to intrinsic biochemical autoregulation than to specific structural outcomes. In this context, both biochemical and morphometric findings are best understood as complementary indicators of tissue stress triggered by amitriptyline exposure, rather than evidence of a mechanistic relationship between a single antioxidant parameter and a defined morphological alteration.

When compared to sibutramine, another serotonin and norepinephrine reuptake inhibitor, amitriptyline demonstrates a distinct mechanism of inducing oxidative stress. The effects of sibutramine are frequently linked to its vasoconstrictive properties, which reduce the supply of nutrients and oxygen to tissues, thereby increasing ROS production [17]. In contrast, amitriptyline primarily induces oxidative stress through mitochondrial dysfunction, leading to more direct and severe tissue damage. This distinction is evident in the pronounced increase in TBARS concentration and the reduction in antioxidant defenses observed in this study. These differences underscore the unique pathways through which these drugs impact cellular redox balance and tissue integrity.

An increase in total salivary protein concentration in amitriptyline‐treated animals may be linked to elevated norepinephrine levels in synaptic clefts, a phenomenon known to enhance protein‐rich saliva secretion [44]. Furthermore, a significant reduction in salivary amylase activity was observed in the treated group, suggesting that amitriptyline may impair the digestive functionality of saliva. This enzymatic decline might reflect alterations in acinar cell function or secretory mechanisms under chronic sympathetic stimulation. Additionally, prolonged norepinephrine elevation could lead to sustained peripheral vasoconstriction, potentially restricting nutrient and oxygen delivery to the salivary glands. These findings were further corroborated by MANCOVA analysis for both glands, which revealed significant differences in oxidative stress markers, particularly in the submandibular gland, and correlations that demonstrate differential activation of compensatory mechanisms. For instance, the lack of significant changes in glutathione (GSH) levels, as evidenced by weak correlations with both TBARS and TEAC, suggests that alternative antioxidant systems may be more relevant in this context. It is important to highlight that changes in salivary composition must be interpreted cautiously in the absence of salivary flow measurements. Although our study detected increased total protein concentration and reduced amylase activity, these parameters alone cannot distinguish between altered glandular secretion and changes in the aqueous fraction of saliva. We intentionally expressed biochemical assays per unit of volume rather than normalizing them to total protein content, as protein normalization in salivary samples can introduce bias due to hydration status and variability in the solvent fraction. For these reasons, salivary flow was not assessed, particularly because anesthesia and pilocarpine‐induced stimulation could markedly alter the physiological secretion profile, making unstimulated saliva collection the only ideal scenario. Consequently, while salivary composition contributes valuable functional context, it should be interpreted alongside, and not independently from, the biochemical and morphological alterations observed in the glands.

This study was conducted in a healthy animal model, free of comorbidities and pre‐existing pathophysiological alterations. In contrast, amitriptyline is administered clinically to patients with psychiatric disorders, whose conditions may involve chronic inflammation, neuroendocrine dysfunction, and established redox imbalances, elements capable of influencing the response to the drug and potentiating or modifying its oxidative effects. In the experimental model, the absence of underlying disease and the strict control of variables such as diet, age, sex, and environment reduce variability and allow the isolation of the drug's pharmacological effects but do not reproduce the full complexity encountered in clinical settings. Additionally, pharmacokinetic and pharmacodynamic differences between species, associated with the route of administration, dose, and exposure time, impact the distribution and bioavailability profile of amitriptyline, making it necessary to exercise caution when directly extrapolating the findings to clinical practice in humans [45].

This study identified significant oxidative and morphological alterations in the salivary glands of rats treated with amitriptyline but has notable limitations. Its findings rely solely on an animal model, which may not fully capture human physiological responses. Additionally, using only a single dose and a fixed exposure period restricts the understanding of dose‐ and time‐dependent effects. The lack of analysis of molecular pathways, such as mitochondrial signaling and apoptosis, further limits the insights. Future research should explore varying doses and exposure durations, assess the reversibility of changes posttreatment, and investigate molecular and proteomic markers to clarify the mechanisms of toxicity and provide a basis for therapeutic strategies that minimize its adverse effects. Additionally, it is important to highlight that the route of administration used in animal models, according to Vandenberg et al. [46], such as gavage, can modify the salivary exposure profile in relation to oral administration in humans, since this method bypasses absorption through the oral mucosa and can result in differences in the absorption and bioavailability profiles of the drug. The choice of gavage allows the direct administration of the drug into the gastrointestinal tract, not allowing direct contact with the salivary glands evaluated, which are located in different anatomical planes of the oral mucosa. Thus, the use of gavage allows greater control of administration and consequently greater bioavailability of the drug.

## 5 Conclusions

Administration of amitriptyline for 30 days resulted in changes in salivary composition, oxidative damage, and morphometric changes in the salivary glands. In addition, the data underscore the relevance of salivary glands as peripheral targets of systemic pharmacological toxicity and highlight the need for further research to investigate the mechanisms involved and possible alterations in humans.

## Author Contributions

Conception, study design, data acquisition, data analysis, and interpretation: Cristian dos Santos Pereira, Deiweson Souza‐Monteiro, Yago Gecy de Sousa Né, Jorddy Neves da Cruz, Vinicius Ruan Neves dos Santos, Everton Luiz Pompeu Varela, Sandro Percário, Leonardo Oliveira Bittencourt, Antonio Hernandes Chaves‐Neto, and Rafael Rodrigues Lima. Writing and critical review of intellectual content: Cristian dos Santos Pereira, Deiweson Souza‐Monteiro, Yago Gecy de Sousa Né, Jorddy Neves da Cruz, Vinicius Ruan Neves dos Santos, Everton Luiz Pompeu Varela, Sandro Percário, Leonardo Oliveira Bittencourt, Antonio Hernandes Chaves‐Neto, and Rafael Rodrigues Lima. Final approval of the version to be submitted: Cristian dos Santos Pereira, Deiweson Souza‐Monteiro, Yago Gecy de Sousa Né, Jorddy Neves da Cruz, Vinicius Ruan Neves dos Santos, Everton Luiz Pompeu Varela, Sandro Percário, Leonardo Oliveira Bittencourt, Antonio Hernandes Chaves‐Neto, and Rafael Rodrigues Lima. All data were internally generated by the research team; no paper mills were involved, and no artificial intelligence tools were used for the creation of text or figures. All authors reviewed and approved the final version of the manuscript. Coordination and supervision: Rafael Rodrigues Lima.

## Ethics Statement

Ethical approval for this study was granted by the Animal Use Ethics Committee (CEUA) of the Federal University of Pará (UFPA), Protocol Number 5458260821.

## Conflicts of Interest

The authors declare no conflicts of interest.

## Supporting information


**Supplementary Table 1:** Description of all analysis values of the study.

## Data Availability

Data generated or analyzed during this research are available from the corresponding author upon reasonable inquiry.
