# Supplementary material for: From Redox Imbalance to Tissue Injury: Insights Into Antidepressant Drug Amitriptyline Effects on Salivary Glands
Source: Cell Biochem Funct. 2026 Mar 1;44(3):e70189. doi: 10.1002/cbf.70189 (PMC12949951; doi:10.1002/cbf.70189)
Supplement: Supplementary file 1 — Supplementary Table 1: Description of all analysis values of the study. [file CBF-44-e70189-s001.docx]

**Supplementary table 1.** Description of all analysis values of the study.

| Variable | Description of unit | Shapiro Wilk W (Control) | Shapiro Wilk W (Amitriptyline) | Shapiro Wilk p (Control) | Shapiro Wilk p (Amitriptyline) | Mean (Control) | Mean (Amitriptyline) | SD (Control) | SD (Amitriptyline) | Power (1-β err prob) |
| --- | --- | --- | --- | --- | --- | --- | --- | --- | --- | --- |
| Salivary amylase | U/dl | 0.9045 | 0.8690 | 0.3589 | 0.1819 | 706.6 | 696.6 | 8.734 | 5.563 | 72.41% |
| Saliva total protein | g/dl | 0.9540 | 0.9688 | 0.7661 | 0.8893 | 0.007 | 0.013 | 0.004 | 0.002 | 94.41% |
| TEAC - Saliva | μmol/L | 0.9708 | 0.9098 | 0.9042 | 0.3945 | 0.098 | 0.095 | 0.029 | 0.036 | 2.88% |
| TEAC – Submandibular | μmol/L | 0.8472 | 0.9779 | 0.1158 | 0.9486 | 0.366 | 0.284 | 0.033 | 0.050 | 95.17% |
| TEAC – Parotid | μmol/L | 0.8344 | 0.8233 | 0.0881 | 0.0691 | 0.242 | 0.150 | 0.066 | 0.031 | 91.59% |
| GSH – Submandibular | μmol/L | 0.8803 | 0.9015 | 0.2276 | 0.3401 | 0.008 | 0.005 | 0.007 | 0.003 | 17.94% |
| GSH – Parotid | μmol/L | 0.8851 | 0.7865 | 0.2501 | 0.0300 | 0.002 | 0.002 | 0.001 | 0.001 | 1.073% |
| TBARS – Submandibular | μmol/L | 0.9668 | 0.8765 | 0.8746 | 0.2114 | 2.497 | 4.269 | 0.326 | 0.676 | 100% |
| TBARS – Parotid | μmol/L | 0.8415 | 0.7702 | 0.1026 | 0.0205 | 2.827 | 5.133 | 0.327 | 0.407 | 100% |
| Total acinar area – Submandibular | µm^2^ | 0.9404 | 0.9584 | 0.6422 | 0.8045 | 63404 | 52831 | 3077 | 3165 | 100% |
| Total ductal area – Submandibular | µm^2^ | 0.8724 | 0.8806 | 0.1948 | 0.2293 | 10185 | 7546 | 560.8 | 1311 | 99.83% |
| Total stromal area – Submandibular | µm^2^ | 0.8502 | 0.8589 | 0.1234 | 0.1480 | 6511 | 12553 | 2164 | 3517 | 97.2% |
| Total acinar area – Parotid | µm^2^ | 0.9759 | 0.9673 | 0.9376 | 0.8785 | 63780 | 63526 | 460.6 | 307.8 | 22.76% |
| Total ductal area – Parotid | µm^2^ | 0.5275 | 0.8780 | 0.0001 | 0.1178 | 3102 | 1352 | 1182 | 323.3 | 96.55% |
| Total stromal area – Parotid | µm^2^ | 0.9039 | 0.8022 | 0.3549 | 0.0431 | 12447 | 15009 | 1723 | 2379 | 63.6% |

Supplementary Table 1: Oxidative biochemistry assays (TEAC: Total Antioxidant Capacity, GSH: Reduced Glutathione Levels, and TBARS: Thiobarbituric acid reactive substances levels) and Morphometric analysis (Total acinar, ductal, and stromal areas) values of the experimental study. Results are expressed as Shapiro-Wilk W and p values, mean, SD: Standard deviation, and Test Power (1-β error probability).
